# Delphinidin sensitizes prostate cancer cells to TRAIL-induced apoptosis, by inducing DR5 and causing caspase-mediated HDAC3 cleavage

**DOI:** 10.18632/oncotarget.3667

**Published:** 2015-03-26

**Authors:** Hyeonseok Ko, Mi-Hyeon Jeong, Hyelin Jeon, Gi-Jun Sung, Youngsin So, InKi Kim, JaeKyoung Son, Sang-wook Lee, Ho-Geun Yoon, Kyung-Chul Choi

**Affiliations:** ^1^ Department of Biomedical Sciences and Department of Pharmacology, University of Ulsan College of Medicine, Seoul, Korea; ^2^ Department of Radiation Oncology, University of Ulsan College of Medicine, Asan Medical Center, Seoul, Korea; ^3^ Asan Institute for Medical Research, University of Ulsan College of Medicine, Asan Medical Center, Seoul, South Korea; ^4^ Department of Biochemistry and Molecular Biology, Brain Korea PLUS Project for Medical Sciences, Yonsei University College of Medicine, Seoul, Korea; ^5^ Laboratory of Molecular Oncology, Cheil General Hospital & Women's Healthcare Center, College of Medicine, Dankook University, Seoul, South Korea

**Keywords:** delphinidin, TRAIL, apoptosis, HDAC3, prostate cancer

## Abstract

TRAIL can induce apoptosis in some cancer cells and is an immune effector in the surveillance and elimination of developing tumors. Yes, some cancers are resistant to TRAIL. Delphinidin, a polyphenolic compound contained in brightly colored fruits and vegetables, has anti-inflammatory, anti-oxidant, and anti-tumorigenic activities. Here we showed that delphinidin sensitized TRAIL-resistant human prostate cancer cells to undergo apoptosis. Cells treated with delphinidin and TRAIL activated the extrinsic and intrinsic pathways of caspase activation. TRAIL-induced apoptosis in prostate cancer cells pretreated with delphinidin was dependent on death receptor 5 (DR5) and downstream cleavage of histone deacetylase 3 (HDAC3). In conclusion, delphinidin sensitizes prostate cancer cells to TRAIL-induced apoptosis by inducing DR5, thus causing caspase-mediated HDAC3 cleavage. Our data reveal a potential way of chemoprevention of prostate cancer by enabling TRAIL-mediated apoptosis.

## INTRODUCTION

Prostate cancer is one of the most frequently diagnosed, non-cutaneous neoplasms in men and the second leading cause of cancer-related mortality among men in the United States. Prostate cancer is unique among human cancers because of its striking age-dependent incidence and variable penetrance. Genetic, epigenetic, and environmental factors, including diet, also contribute to the prostate carcinogenesis process [1, 2]. In other words, the development of prostate cancer in humans presents as a multistage process, involving a small latent carcinoma of low histological grade at its onset, which later progresses to a large metastatic lesion [3]. However, therapeutic strategies for this disease are limited because chemotherapy and radiation therapy are largely ineffective, and metastatic disease frequently develops even after potentially curative surgery [4-6]. Therefore, developing a novel therapeutic strategy for prostate cancer has become an important medical need.

Tumor necrosis factor - related apoptosis - inducing ligand (TRAIL) is a member of the tumor necrosis factor (TNF) family of cytokines. TRAIL exerts proapoptotic effects on malignant cells without any harmful effects to normal cells. Endogenous TRAIL triggers death signaling via receptor-mediated apoptosis through its interaction with these death receptors (DRs) on the surface of cancer cells [7]. Five members of the death receptor family have been identified that can bind TRAIL. The death receptors, DR4 (TRAIL-R1) and DR5 (TRAIL-R2), contain both two cysteine-rich extracellular TRAIL-binding domains and a cytoplasmic death domain that are required for transmitting a cytotoxic signal [8, 9]. The decoy receptors, DcR1 (TRAIL-R3) and DcR2 (TRAIL-R4), also possess binding ability for TRAIL, but they do not transmit apoptotic signals due to a nonfunctional death domain [10, 11]. Finally, TRAIL initiates programmed cell death upon binding to DR4 and/or DR5, which induces clustering of the bound DRs (forming a microaggregate within the cell membrane), and promotes the recruitment of the adaptor molecule FADD (FAS-associated death domain). This results in the formation of DISC (death inducing signaling complex) and subsequent effector caspases. TRAIL-mediated apoptosis can also be induced by the intrinsic pathway, with the implication of mitochondrial dysfunction, and the extrinsic pathway [12]. Caspase-8, which contains a death effector domain, is activated by a death receptor signaling pathway, and caspase-9, which contains a caspase activation and recruitment domain (CARD), is activated by a mitochondrial death signaling pathway [13]. The link between the extrinsic and intrinsic signaling pathways is formed by the BID (BH3-interacting domain death agonist) protein, which is cleaved and activated by caspase-8. Then, active caspase 8 cleaves and directly activates downstream effector caspases (3, 6 and 7), which ultimately cut vital cellular substrates and cause apoptosis [14, 15]. BCL-2 family proteins, which comprise both anti-apoptotic members such as BCL-2 and MCL-1 and pro-apoptotic molecules such as BAX, play an important role in the regulation of the mitochondrial apoptotic pathway [16]. TRAIL treatment results in up-regulation of DR5 expression, activation of caspase-8 and BID, leading to BAX conformational changes and translocation to the mitochondria. This, in turn, causes a loss of mitochondrial membrane potential and release of cytochrome c into the cytoplasm, followed by activation of caspase-3, and induction of caspase-dependent apoptosis [17]. Inhibitor of Apoptosis proteins (IAPs) can block executioner caspases. Specifically, XIAP and survivin are potent inhibitors of caspase 9, while cIAP-2 and XIAP can inhibit caspases 3 and 7 [18, 19]. p53 can act in both the extrinsic pathway to up-regulated TRAIL receptors directly in a p53-dependent manner or to activate pro-apoptotic elements, such as BAX through the intrinsic apoptotic pathway [20]. More recently, enhancement of p53 acetylation levels strongly correlates with protein stabilization and activation in response to cellular stress and is indispensable for p53 transcriptional activity [21, 22].

However, some tumor cells - including prostate cancer and gliomas - are resistant to TRAIL-induced apoptosis [23-26]. Failure to undergo apoptosis has been implicated in the resistance of cancer cells to TRAIL surveillance, and, therefore, in tumor development [14]. In addition, an obstacle to effective therapy is that prostate cancer, similar to many other cancers, develops resistance to TRAIL [27-29]. Thus, researchers are currently seeking to identify TRAIL sensitizers which is capable of overcoming TRAIL resistance in cancer cells. Novel agents, such as natural compounds, are needed to overcome this resistance and to improve TRAIL efficacy. Recently, a variety of agents, such as various types of isoflavones [30], ursolic acid [31], carnitine [32], salirasib [33], monensin [34], and 2-tellurium-bridged β-cyclodextrin [35], have been reported to sensitize tumors to TRAIL-induced apoptosis. Recently, cancer prevention research reports have shown that chemopreventive agents, such as curcumin, EGCG (epigallocatechin gallate), and resveratrol, have the therapeutic potential to sensitize prostate cancer cells to TRAIL [36-38]. In this study, we examined this hypothesis to determine whether delphinidin enhances the therapeutic potential of TRAIL and induces apoptosis of TRAIL-resistant prostate cancer LNCaP cells.

Furthermore, resistance to TRAIL is an important therapeutic problem that may be resolved by combination treatments, but it act by various mechanisms, including the restoration of caspase-8 expression or decrease in c-FLIP levels [39, 40]. Because most agents used in such combinations are inherently toxic, it is imperative to find nontoxic agents, such as HDAC inhibitors (HDACi), which have recently entered clinical trials and exert their antitumor effects by inducing growth arrest, differentiation, and apoptosis [41-44]. To date, eighteen distinct HDACs have been identified and classified into four groups based on their structural divergence, namely, class I, II, III, and IV HDACs [45, 46]. There are three classes of mammalian HDAC enzymes, class I comprising HDAC1, HDAC2, HDAC3, and HDAC8; class II HDACs comprising HDAC4, HDAC5, HDAC6, HDAC7, HDAC9, and HDAC10; and a third class of NAD-dependent SIR2 deacetylases [43-45]. These HDACs are involved in modulating most key cellular processes, including apoptosis, autophagy, transcriptional regulation, metabolism, DNA damage repair, cell cycle control, senescence, and chaperone function [47]. However, HDACs have been found to function incorrectly in cancer. Therefore, HDAC inhibition by an HDAC inhibitor or by HDAC cleavage has been explored as a chemotherapeutic strategy to interfere with aberrant HDAC activity in human cancers. Several studies have demonstrated that a combination strategy of an HDAC inhibitor with a TRAIL sensitizer has emerged as a potent strategy to prime cancer cells to TRAIL-mediated apoptosis in a variety of human cancers [48]. Recently, HDAC cleavage has been shown to play an important role in apoptosis in response to various cellular stimuli. For example, the apoptosis induced by cleaved HDAC3 or enhanced by c-JUN deficiency during osmotic stress is suppressed by exogenous expression of c-JUN, indicating that the down-regulation of c-JUN by HDAC3-dependent transcriptional repression plays a role in regulating cell survival and apoptosis [49]. Caspase-dependent HDAC3 degradation, which is involved in the regulation of E2F-1 transcription, has also been observed in neuronal apoptosis [50]. Notably, *Fabrice E* and *colleagues* demonstrated the role of HDAC3 in apoptosis control. They showed that C-terminal cleavage of HDAC3 is caspase dependent and accumulates in the cytoplasm. The cleavage and cytoplasm re-localization of HDAC3 caused by apoptosis stimuli induces apoptosis of cells via the activation of proapoptotic genes and the inhibition of anti-apoptotic genes [51]. Thus, cleaved HDAC3 is indispensable for inducing cell apoptosis.

Anthocyanins are naturally occurring flavonoids that are responsible for the bright colors of many fruits and vegetables. Anthocyanins are organic compounds, which are derivatives of the glycosylation of aglycon anthocyanidin, and more than 500 kinds of compounds, with differences in the number of added sugars, are estimated to exist. As representatives of anthocyanidins, delphinidin, pelargonidin, cyanidin, and malvidin are naturally occurring [52-54]. Delphinidin, one of the major anthocyanidins present in these fruits and vegetables, is a diphenylpropane-based polyphenolic ring structure that carries a positive charge on its central ring [55]. Delphinidin possesses anti-oxidant [56], anti-inflammatory [57], anti-angiogenic [58] and anti-mutagenic activity [59], and was recently reported to inhibit invasion of breast cancer cells [60]. Other studies have revealed that delphinidin inhibits proliferation and induces apoptosis in many different cancer models including colon, uterine, breast, and prostate [61-64]. However, dd effects of delphinidin on TRAIL-induced apoptosis and the underlying molecular mechanisms for those effects in prostate cancer cells.

In this study, we demonstrated that delphinidin potently sensitized human prostate cancer cells to TRAIL-mediated apoptosis via DR5 induction and the caspase-dependent pathway. Furthermore, we showed for the first time that cleavage of HDAC3 had a critical role in this caspase-dependent apoptotic pathway on TRAIL-induced apoptosis in the presence of delphinidin. Therefore, The combination delphinidin with TRAIL could be attractive strategy for the treatment of TRAIL-resistant prostate cancer.

## RESULTS

### Delphinidin enhances TRAIL-mediated apoptosis in prostate cancer cells

**Figure 1:**
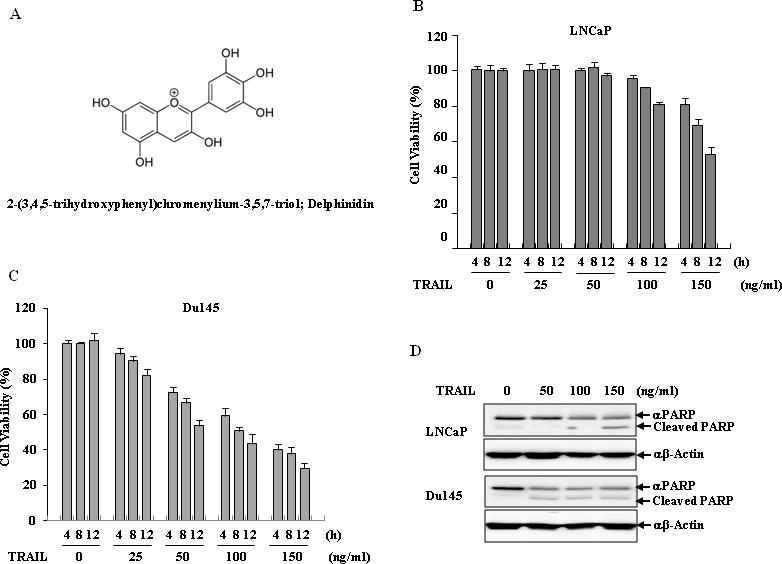
Delphinidin sensitizes TRAIL-mediated apoptosis in human prostate cancer cells (**A**) The chemical structure of delphinidin. (B and C) Anti-proliferation effect of TRAIL in human prostate cancer cell lines. LNCaP (**B**) and Du145 cells (**C**) were treated with TRAIL in a dose- and time-dependent manner. LNCaP and Du145 cells were treated with different concentrations of TRAIL (0, 25, 50, 100, 150 ng/ml) for the indicated times (4, 8, 12 h) and cell viability was measured by MTT assay. The data are expressed as the mean ± SD for triplicates. (**D**) TRAIL induced apoptosis in prostate cancer cell lines. LNCaP and Du145 cells were treated with different concentration of TRAIL (0, 50, 100, 150 ng/ml) for 12 h. Cell viability was measured by a MTT assay.

LNCaP cells are more refractory to TRAIL-induced apoptosis than Du145 cells. Using the MTT assay and western blot analysis to assess PARP cleavage, we confirmed this differential sensitivity to the anti-proliferative effects and apoptosis in a dose- and time-dependent manner, respectively. As shown in Fig. 1B and 1C, TRAIL treatment for 12 h LNCaP cells were refractory to a TRAIL-induced anti-proliferative effect to a dose as high as 100 ng/ml, while treatments with 50 ng/ml TRAIL resulted in approximately 50% inhibition of cell growth in Du145 cells. Apoptosis was activated in both LNCap and Du145 cells upon treatment with 150 ng/ml and 50 ng/ml of TRAIL for 12 h, respectively, as confirmed by the results for PARP cleavage (Fig. 1D).

**Figure 2:**
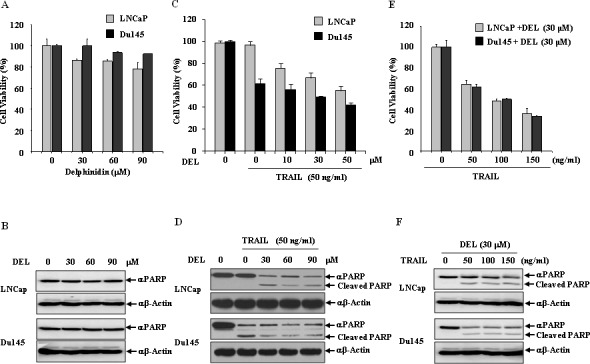
Delphinidin sensitizes LNCaP and Du145 cells with TRAIL-mediated apoptosis (**A** and **B**) Delphinidin has anti-proliferative and proapoptotic effects in prostate cancer cells. LNCaP and Du145 cells were treated with different concentration of delphinidin (DEL) (0, 30, 60, 90 μM) for 12 h. Delphinidin had no effect on cell viability at these concentrations as measured by an MTT assay (A) and western blot analysis against apoptosis marker PARP showed no cleavage (**B**). (C–F) Delphinidin sensitized LNCaP and Du145 cells to TRAIL-mediated apoptosis. LNCaP and Du145 cells were treated with different concentrations of delphinidin (0, 10, 30, 50 μM) and/or a fixed concentration of TRAIL (50 ng/ml) for 12 h. Combined treatment increased cell death as measured by an MTT assay (**C**) and PARP cleavage (**D**). Subsequently, LNCaP and Du145 cells were treated with different concentrations of TRAIL (0, 50, 100, 150 ng/ml) and a fixed concentration of delphinidin (30 μM) for 12 h. Combined treatment increased cell death (**E**) and PARP cleavage (**F**). The data are expressed as the mean ± SD for triplicates.

We first measured the effect of delphinidin on cell viability and PARP cleavage using western blot analysis in human prostate cancer cell lines. We examined whether delphinidin induced apoptosis in LNCaP and Du145 cells. Cells were treated with various low-dose concentrations (0-90 μM) of delphinidin for 12 h. We then observed that low-dose delphinidin did not inhibit cell viability (Fig. 2A) and PARP cleavage (Fig. 2B) in LNCaP and Du145 cells, respectively. Next, we examined the effect on cell viability and PARP cleavage of combining delphinidin (0-30 μM) with 50 ng/ml TRAIL. Delphinidin strongly synergized with TRAIL to induce an anti-proliferative effect in a dose-dependent manner (Fig. 2C). As shown in Fig. 2D, in TRAIL-resistant LNCaP cells no cleavage of PARP occurred upon treatment with 50 ng/ml TRAIL alone, but TRAIL treatment cleaved PARP in the presence of 10 μM delphinidin. In contrast, in TRAIL-sensitive Du145 cells PARP cleavage was induced by TRAIL treatment even in the absence of delphinidin. To further investigate the anti-proliferative and proapoptotic effects of delphinidin, we examined whether delphinidin could sensitize LNCaP and Du145 cells to TRAIL-mediated cell growth inhibition and to induce apoptosis. LNCaP and Du145 cells were treated for 12 h with delphinidin (30 μM) along with various concentrations of TRAIL. Fig. 2E shows that after 12 h delphinidin treatment synergistically sensitized the anti-proliferative effect in response to TRAIL. The co-treatment with delphinidin (30 μM) and various concentrations of TRAIL similarly induced PARP cleavage in TRAIL-sensitive Du145 cells and TRAIL-resistant LNCaP cells (Fig. 2F). These results suggest that delphinidin increases the apoptotic ability of TRAIL in prostate cancer cells. In subsequent experiments, we used TRAIL-resistant LNCaP cells to reveal the delphinidin-induced sensitization mechanisms of TRAIL-mediated apoptosis.

### Delphinidin accelerates TRAIL-induced apoptosis by activating a caspase-dependent pathway in LNCaP cells

**Figure 3:**
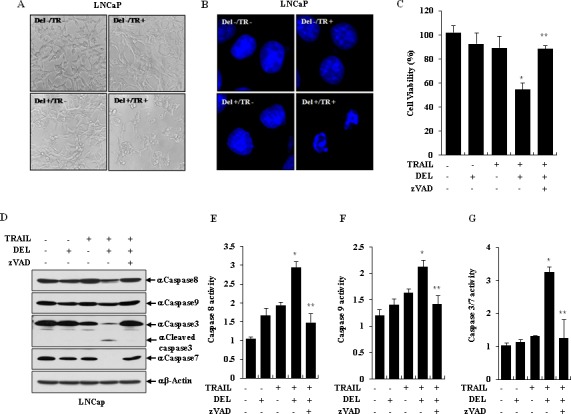
TRAIL and delphinidin induces apoptosis via activation of caspases in LNCaP cells (**A** and **B**) LNCaP cells were treated with TRAIL (TR, 50 ng/ml) and/or delphinidin (DEL, 30 μM) for 12 h. (A) The cell morphology was visualized by an inverted microscope (magnification ×100). (B) By DAPI staining, the cells clearly show condensed chromatin or fragmented nuclei which were taken as apoptotic bodies (magnification ×1000). (**C** and **D**) TRAIL-mediated apoptosis with delphinidin was suppressed by caspase inhibitor zVAD. LNCaP cells were pretreated for 30 min with or without zVAD (40 μM) before TRAIL (50 ng/ml) and/or delphinidin (30 μM) treatment for 12 h. The increased apoptosis from combination treatment was blocked by zVAD (C) and the levels of caspase-8, caspase-9, and cleaved caspase-3 and caspase-7 were reduced by zVAD treatment on western blot analysis (D). (**E**-**G**) TRAIL and delphinidin dramatically induced activation of caspases in LNCaP cells. LNCaP cells were pretreated for 30 min with or without zVAD (40 μM) before TRAIL (50 ng/ml) and/or delphinidin (30 μM) treatment for 12 h. After incubation, the activities of caspase-3/7, caspase-8, and caspase-9 were inhibited by zVAD treatment. All data are expressed as the mean ± SD for triplicates (*P<0.05 vs. −TRAIL/−DEL; **P<0.05 vs. with +TRAIL/+DEL).

To observe whether the combination of delphinidin with TRAIL leads to activation of apoptosis in TRAIL-resistant LNCaP cells, apoptotic cell death and nuclear fragmentation/shrinkage was monitored by cell morphology and DAPI staining. As shown in Fig. 3A, the morphology of LNCaP cells was significantly altered by the combination of TRAIL with delphinidin, but not by either individual treatment. The nuclear morphology in DAPI-stained cells was destroyed during TRAIL-mediated apoptosis in the presence of delphinidin (Fig. 3B).

To further explore the apoptosis-inducing mechanism, we examined the activation and expression of caspase proteins. Preferentially, to determine whether combined treatment with delphinidin and TRAIL induces caspase-dependent apoptosis, LNCaP cells were incubated for 12 h with 50 ng/ml TRAIL and 30 μM delphinidin in the presence or absence of zVAD, a general caspase inhibitor. As shown in Fig. 3C, treatment with zVAD remarkably reduced the ability of delphinidin to sensitize LNCaP cells to TRAIL-induced apoptosis. As expected from the zVAD antagonism, co-treatment with TRAIL and delphinidin induced a significant cleavage of caspase-3 and caspase-7 on western blot analysis (Fig. 3D). The levels of cleaved initiator caspase-8 and caspase-9 were enhanced by co-treatment with TRAIL and delphinidin, and were completely inhibited by zVAD (Fig. 3D). As shown in Fig. 3E-G, treatment with zVAD dramatically repressed the activity of caspase-3/7, caspase-8 and caspase-9 in LNCaP cells and attenuated the sensitization induced by the combined treatment with TRAIL and delphinidin.

### TRAIL-induced apoptosis in cells pretreated with delphinidin involves both the DR5 and the intrinsic apoptotic pathways

**Figure 4:**
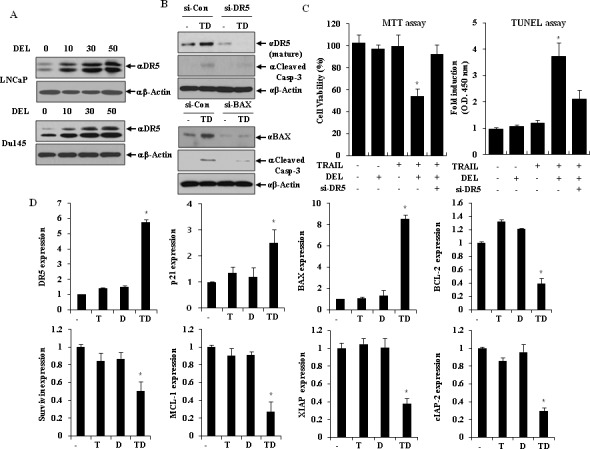
TRAIL and delphinidin activate DR5 expression and intrinsic apoptotic pathway (**A**) Delphinidin induces DR5 in LNCaP and Du145 cells. LNCaP and Du145 cells were treated for 12 h with different concentration of delphinidin (0, 10, 30, 50 μM), and western blot analysis was performed using antibodies against DR5. (B and C) DR5 and BAX expression is critical for the sensitization of TRAIL-mediated apoptosis. LNCaP cells transfected with the scrambled siRNA, DR5 siRNA, or BAX siRNA were further treated with 30 μM delphinidin and 50 ng/ml TRAIL for 12 h. (**B**) Western blot analysis demonstrated inhibition of caspase-3 cleavage by knockdown of DR5 or BAX and (**C**) their corresponding antiapoptotic effects. The apoptotic cell death was determined by MTT assay (left) and TUNEL assay (right) kit as described in Materials and Methods. (**D**) Co-treatment of delphinidin and TRAIL regulates the expression of various apoptosis-related genes at transcriptional level. LNCaP cells were treated with TRAIL (T, 50 ng/ml) and/or delphinidin (D, 30 μM) for 12 h. The expression level of each gene was analyzed by qRT-PCR using total mRNA from LNCaP cells, treated with delphinidin and/or TRAIL, and compared with control LNCaP cells. Fold-change was calculated by 2^−ΔΔCt^ relative quantitative analysis. The data are expressed as the mean ± SD for triplicates (*P<0.05 vs. −TRAIL/−DEL).

Because DR5 on the cell surface trigger apoptosis dependent on TRAIL binding, we investigated whether delphinidin induced DR5 protein expression in a dose-dependent manner in LNCaP and Du145 cells. As shown in Fig. 4A, delphinidin treatment up-regulated the expression of DR5 protein in a dose-dependent manner, siRNA-mediated suppression of DR5 effectively blocked delphinidin-stimulated TRAIL-induced caspase-3 activation (Fig. 4B, upper) and apoptosis (Fig. 4C). Next, we investigated whether the modulation of BAX proteins is involved in the sensitization of LNCaP cells to TRAIL-induced apoptosis by delphinidin. Co-treatment of LNCaP cells with delphinidin and TRAIL resulted in the up-regulation of BAX expression, and siRNA-mediated knock-down of BAX markedly inhibited delphinidin-stimulated TRAIL-induced caspase-3 activation (Fig. 4B, bottom). Because the combined treatment with TRAIL and delphinidin leads to the modulation of BCL-2 family proteins related to the intrinsic apoptotic pathway - including the IAPs, MCL-1, and p21 - we evaluated the mRNA expression of these proteins in LNCaP cells. As shown in Fig. 4D, co-treatment resulted in the up-regulation of BAX and p21 as well as DR5 in LNCaP cells; whereas, it significantly reduced the mRNA expression level of XIAP, cIAP-2, Bcl-2, survivin and MCL-1.

### HDAC3 cleavage is critical for the regulation of apoptosis-related proteins in delphinidin-stimulated TRAIL-mediated apoptosis

**Figure 5:**
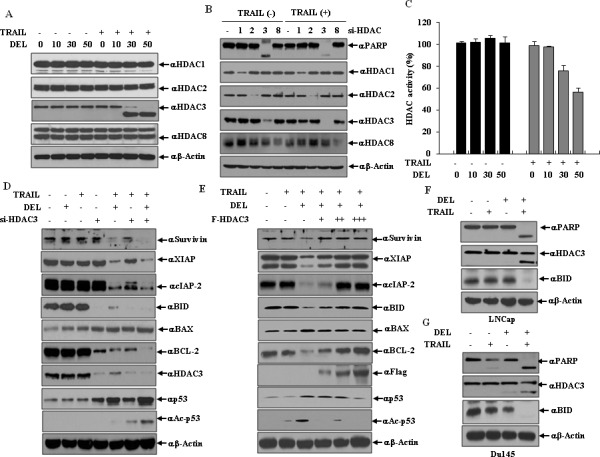
HDAC3 cleavage contributes to the regulation of apoptosis-related proteins on co-treatment delphinidin and TRAIL (**A**) HDAC3 is cleaved by co-treatment of delphinidin and TRAIL. LNCaP cells were incubated with different concentration of delphinidin (0, 10, 30, 50 μM) with or without TRAIL (50 ng/ml) for 12 h. Western blot analysis was performed by using antibodies against HDAC1, HDAC2, HDAC3 or HDAC8. (**B**) Knockdown of HDAC3 sensitizes to TRAIL. LNCaP cells transfected with the indicated siRNA (scrambled siRNA, *HDAC1*, *HDAC2*, *HDAC3*, or *HDAC8*). Following transfection, cells were also exposed for a further 12 h to TRAIL (50 ng/mL), and analyzed by western blot with anti-HDAC1, HDAC2, HDAC3, HDAC8, or PARP antibodies. (**C**) Down-regulation of HDAC activity is induced by co-treatment of delphinidin and TRAIL. LNCaP cells were incubated with different concentrations of delphinidin (0, 10, 30, 50 μM) with or without TRAIL (50 ng/ml) for 12 h. After incubation, HDAC activity was measured by an HDAC activity ELISA assay kit. All data are expressed as the mean ± SD for triplicates. (D and E) HDAC3 regulates the expression of apoptosis-related proteins during co-treatment with delphinidin and TRAIL. (**D**) LNCaP cells were transfected with HDAC3 siRNA or a scrambled siRNA. Following transfection, cells were also exposed for a further 12 hours to delphinidin (30 μM) and/or TRAIL (50 ng/mL), and analyzed by western blot analysis with survivin, XIAP, cIAP-2, BID, BAX, BCL-2, HDAC3, p53, and acetylated p53 (Ac-p53) antibodies. (**E**) LNCaP cells were transfected with the empty plasmid or HDAC3 expression plasmid (*F-HDAC3*). Following transfection, cells were also exposed for a further 12 h to delphinidin (30 μM) and/or TRAIL (50 ng/mL), and the apoptotic factors above were analyzed by western blot analysis. (**F** and **G**) Combined treatment with TRAIL and delphinidin leads to apoptosis and cleavage of HDAC3 in prostate cancer cell lines. LNCaP (F) and Du145 cells (G) were treated with TRAIL (50 ng/ml) and/or delphinidin (30 μM) for 12 h, and then a western blot analysis was performed using PARP, HDAC3, or BID antibodies.

The epigenetic changes of apoptosis are attended by chromatin condensation and chromatin breaks. As shown in Fig. 5A, combined treatment with delphinidin and TRAIL resulted in the down-regulation of HDAC3 expression which is proportional to an increasing concentration of delphinidin, but neither delphinidin nor TRAIL alone down-regulated HDAC3 expression. In contrast, the expression levels of other class I HDACs HDAC1, HDAC2 and HDAC8, were not altered. As shown in Fig. 5B, siRNA-mediated knockdown of HDAC3 expression induced PARP cleavage the presence of TRAIL while HDAC3 silencing induced PARP cleavage to a lesser degree also in the absence of TRAIL. However, siRNA-mediated knockdown of other class I HDACs had no effect PARP cleavage regardless of treatment with TRAIL. HDAC3 activity (Fig. 5C) was also reduced by co-treatment with TRAIL and delphinidin. In the combined treatment with delphinidin and TRAIL, siRNA-mediated suppression of HDAC3 more effectively blocked the expression of survivin, BCL-2, XIAP, BID, and cIAP-2, and increased the expression of BAX compared with siRNA HDAC3 alone (Fig. 5D). Also, interestingly, the levels of acetylated p53 and total p53 increased more markedly in the combined treatment with delphinidin and TRAIL compared with either alone Fig. 5D. Conversely, exogenously expressed HDAC3 increased the expression of antiapoptotic factors survivin, BCL-2, XIAP, BID and cIAP-2 in a dose-dependent fashion, and remarkably decreased the expression of proapoptotic factors BAX, p53 and acetylated p53, antagonizing the negative and positive effects of the delphinidin plus TRAIL combination (Fig. 5E). Additionally, combined treatment with TRAIL and delphinidin led to apoptosis and cleavage of HDAC3 in TRAIL-resistant LNCaP cells as well as TRAIL-sensitive Du145 cells (Fig. 5F and 5G). Taken together, these results demonstrate that the cleavage of HDAC3 contributes to the sensitizing effect of delphinidin on TRAIL-mediated apoptosis, which involves the regulation of mitochondrial proteins, IAPs, p53, and acetylated p53.

### HDAC3 cleavage is regulated by DR5-mediated effector caspases activation and accelerated the delphinidin-induced sensitization of TRAIL-mediated apoptosis

**Figure 6:**
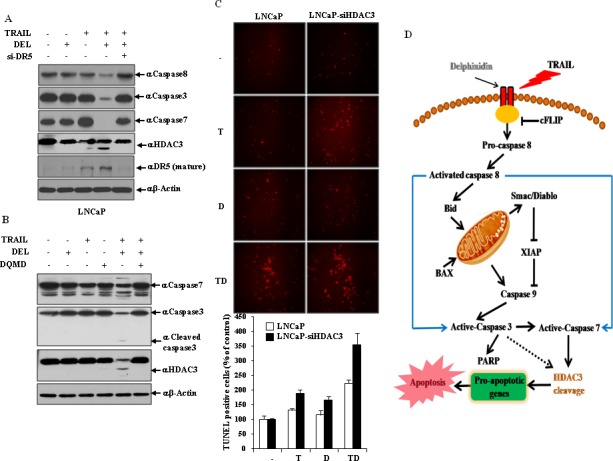
HDAC3 cleavage is regulated by DR5-mediated effector caspases activation in the delphinidin-induced sensitization of TRAIL-mediated apoptosis (**A**) DR5 expression is critical for the activation of caspases and cleavage of HDAC3 in the co-treatment of delphinidin and TRAIL. LNCaP cells were transfected with the scrambled siRNA or DR5 siRNA. Following transfection, cells were incubated for a further 12 h with delphinidin (30 μM) and/or TRAIL (50 ng/mL), and analyzed by western blot analysis with caspase-8, caspase-3, caspase-7, and HDAC3 antibodies. (**B**) Cleavage of HDAC3 depends on the activation of effector caspases. LNCaP cells were pretreated for 30 min with or without caspase-3/7 inhibitor DQMD (20 mM) before TRAIL (50 ng/ml) and/or delphinidin (30 μM) treatment for 12 h. After incubation, western blot analysis was performed by using antibodies against caspase-7, caspase-3, cleaved caspase-3, and HDAC3. (**C**) TUNEL assay indicating the additive effects of TRAIL and delphinidin on induction of apoptosis in LNCaP cells (*left*). TUNEL-positive apoptotic cells were counted in five different high power fields (*right*). The data are expressed as percent TUNEL-positive cells. The data are expressed as the mean ± SD for triplicates. (**D**) Schematic diagram of apoptotic pathway induced by the combined treatment with TRAIL and delphinidin.

As shown in Fig. 6A, in the combined treatment with delphinidin and TRAIL DR5 siRNA more effectively inhibited the activation of effector caspases (caspase-3 and -7) and the cleavage of HDAC3 compared with the scrambled siRNA. Therefore, this result demonstrated that activity of effector caspases and HDAC3 cleavage is altered by DR5, which plays a critical role in the delphinidin-induced sensitization of TRAIL-mediated apoptosis. Because HDAC3 is caspase-dependent in some cell types, we examined the possible involvement of active effector caspases in HDAC3 cleavage. Pretreatment with the caspase 3/7 inhibitor DQMD inhibited the delphinidin and TRAIL-induced activation of caspase 3/7 and remarkably blocked truncation of HDAC3 (Fig. 6B), suggesting that HDAC3 is cleaved by effector caspases during co-treatment. DNA fragmentation represents a characteristic hallmark of apoptosis. TUNEL assay is an established method for detecting DNA fragments. To investigate whether combined treatment of delphinidin and TRAIL can induce apoptotic cell death through HDAC3, TUNEL staining was performed. As shown in Fig. 6C, apoptotic cell death by delphinidin and TRAIL combination treatment was significantly increased by HDAC3 siRNA (3.5-fold) compared with the scrambled siRNA (2.1-fold), suggesting that cleavage of HDAC3 accelerates the sensitizing effect of delphinidin on TRAIL-mediated apoptosis.

## DISCUSSION

TRAIL, also known as APO2L, is a member of the TNF family of death receptor ligands and has significant potential for use in cancer therapy because of its potent ability to selectively kill cancer cells while leaving normal cells unharmed [65]. The selectivity of TRAIL to trigger apoptosis in cancer cells has led to the clinical development of recombinant TRAIL and TRAIL-receptor agonists as an anticancer therapy. TRAIL-based therapies are now in Phase I and II clinical trials (www.clinicaltrials.gov). However, TRAIL will probably not be viable as a single agent since the majority of tumor cells are resistant to TRAIL. Combination therapy (with chemotherapy or radiation) is therefore essential for the use of TRAIL against refractory tumors.

In our current report, we find for the first time that delphinidin, a major anthocyanidin, is capable of sensitizing highly resistant prostate cancer cells to TRAIL-mediated apoptosis. Caspase-dependent pathways of apoptosis are involved in this process, as evidenced by a strong induction of caspase-3/7, caspase-8 and caspase-9 activity. This finding suggests that prostate cancer cells are capable of amplifying apoptotic signaling initiated by DR's through the recruitment of the mitochondrial pathway, as observed in other cell types. Given that TRAIL is known to trigger apoptosis through binding to its cell surface death receptors, DR4 and DR5, the expression levels of these death receptors may be critical to determining the intensity and/or duration of TRAIL-induced apoptotic signaling [8, 9, 66]. We recently showed that the DR5 up-regulating agent, neobavaisoflavone, effectively stimulates TRAIL-mediated apoptosis in prostate cancer cells via the intrinsic pathway [67]. In this study, we find that delphinidin increases the protein level of DR5 in prostate cancer cells. A siRNA-mediated DR5 knockdown effectively inhibits the apoptosis induced by delphinidin plus TRAIL, confirming the functional significance of DR5 up-regulation in delphinidin-stimulated TRAIL-mediated apoptosis. In addition, combined treatment with delphinidin and TRAIL transcriptionally regulates expression of anti-apoptotic and pro-apoptotic molecules as well as DR5. Notably, co-treatment with delphinidin and TRAIL significantly reduced the mRNA expression level of MCL-1, XIAP and cIAP-2. Human MCL-1 is an anti-apoptotic member of the BCL-2 protein family with similar BH (BCL-2 homology)-multidomain structures as BCL-2 and BCL-XL [16, 68]. Recently, it has been reported that increased MCL-1 expression likely confers additional protection against tBID in TRAIL-treated BAX-deficient cells [19]. It follows that in cells with elevated MCL-1 expression, tBID is bound by MCL-1 [69], and all of tBID's proapoptotic activities are inhibited. Thus, we speculate that down-regulation of MCL-1 by delphinidin plus TRAIL might play a critical role in delphinidin-stimulated TRAIL-mediated apoptosis. Also, the potent inhibitors of caspases, XIAP, cIAP-2, and survivin, are down-regulated by delphinidin plus TRAIL. Furthermore, it is reported that salirasib, an S-farnesyl cysteine analog, reduces survivin mRNA expression in human hepatocarcinoma cell lines, and protein expression of survivin markedly decreases after 48 h of treatment with 150 mM of salirasib, coinciding with the beginning of TRAIL sensitization. It is therefore tempting to speculate that salirasib-induced inhibition of survivin has an important role in this process [33]. Therefore, our data suggest that sensitization of LNCaP cells to TRAIL-induced apoptosis by delphinidin occurs through DR5 up-regulation and caspase-dependent mitochondrial pathways (Fig. 6D).

HDAC3 is an important component of nuclear receptor co-repressor (N-CoR)-silencing mediator for retinoid and thyroid receptor (SMRT) co-repressor complexes that include G protein suppressor pathway 2 (GPS2), transducin β-like 1 (TBL1), and transducin β-like X-linked receptor 1 (TBLR1) [70]. We find that cleavage of HDAC3 contributes to the sensitizing effect of delphinidin on TRAIL-mediated apoptosis involving the regulation of mitochondrial proteins. HDAC3 is cleaved, and cleaved HDAC3 localizes to the cytoplasm and can no longer deacetylate histones on the promoters of its proapoptotic targets upon apoptosis induction. Histones on these promoters can become hyperacetylated, thereby allowing the transcriptional activation of these proapoptotic genes and cell progression into apoptosis [51]. Truncated HDAC3, which is generated by caspase-7 cleavage during the early phase of osmotic stress, binds to the *c-JUN* promoter region in a c-JUN-dependent manner, deacetylates histones, and thereby represses *c-JUN* transcription. The abrogated c-JUN expression, which down-regulates c-JUN activation occurring during the early stage of exposure to stress, promotes osmotic stimulation-induced cell apoptosis [49]. Thus, we speculate that delphinidin-stimulated TRAIL-mediated apoptosis is accelerated by the transcriptional activation of proapoptotic genes via cleavage of HDAC3. In addition, we reveal that co-treatment with delphinidin and TRAIL induces DR5 up-regulation and downstream cleavage of HDAC3 by effector caspases. Similar to the repression of *c-JUN* by cleaved HDAC3, HDAC4 is cleaved in a caspase-3-dependent manner [71, 72], causing the N-terminal fragment of HDAC4 to translocate into the nucleus and to represses transcription of myocyte enhancer factor-2C (*MEF2C*) [72]. We believe that the consistency of mechanistic action supports our model suggesting that HDAC3 cleavage by the effector caspases critically contributes to delphinidin-facilitated TRAIL-mediated apoptosis.

In conclusion, we demonstrate for the first time that a combination of delphinidin and TRAIL can effectively induce apoptosis in prostate cancer cells. Also, we find that delphinidin-mediated TRAIL sensitization in prostate cancer cells seem to be associated with activation of effector caspases via induction of the DR5 pathway, leading to HDAC3 cleavage-dependent mitochondrial apoptosis. Therefore, our study provides a possible therapeutic application of delphinidin and TRAIL for treatment of prostate cancers that are resistant to TRAIL.

## MATERIALS AND METHODS

### Cell culture, reagents, and antibodies

Human prostate LNCaP and Du145 cells were obtained from the American Type Culture Collection (Manassas, VA) and cultured in RPMI supplemented with 10% fetal bovine serum (FBS; Gibco-BRL, MD) and 1% antibiotic-antimycotic solution in a humidified 5% CO_2_ atmosphere at 37°C. Antibodies against cleaved caspase-3, caspase-7, caspase-8, caspase-9, PARP, Survivin, XIAP, cIAP-2, BID, BAX, DR5, BCL-2 and acetylated p53 (Ac-p53) antibodies were purchased from Cell Signaling Technology (Beverly, MA). Anti-HDAC1, HDAC2, HDAC3, HDAC8, FLAG, p53, and actin antibodies were purchased from Santa Cruz Biotechnology (Dallas, TX). Anti-FLAG and β-actin antibodies were purchased from Sigma-Aldrich (St. Louis, MO). The anti-mouse or anti-rabbit secondary antibodies were purchased from Pierce (Rockford, IL). Delphinidin and TRAIL were obtained from Sigma-Aldrich (Fig. 1A).

### Caspase assays

Delphinidin and TRAIL-mediated caspase activation was evaluated using Caspase-Glo 3/7, 8, and 9 kits (Promega, Madison, WI) according to the manufacturer's instructions. Briefly, LNCaP cells were plated in 96-well clear-bottom plates (Lonza, Basel, Switzerland). The cells were treated with TRAIL with/without delphinidin. After 24 h, assay reagent (100 μl) was added to each well. The plate was incubated in the dark for 30–60 min, and luminescence was measured using a SpectraMAX 250 Optima plate reader (Molecular Device Co., Sunnyvale, CA).

### Western blot analyses

Western blot analysis was carried out as previously described [73]. Protein extracts were prepared from TRAIL with/without delphinidin treated cells, and the indicated proteins in the figures were determined using western blot analyses. LNCaP cells were treated with TRAIL with/without delphinidin. Cells were collected 24 h after treatment, washed once with PBS, and extracts were prepared with lysis buffer [50 mM Tris-Cl (pH 7.5), 150 mM NaCl, 1% NP40, 10 mM NaF, 10 mM sodium pyrophosphate, and protease inhibitors]. Protein extracts were separated using 10% SDS–polyacrylamide gels, and transferred to nitrocellulose membranes. Blots were blocked for 1 h and then incubated with the primary antibody for 2 h at room temperature or overnight at 4°C, and processed with HRP-conjugated secondary antibody. Protein bands were visualized using film developer.

### MTT assays to measure cytotoxicity

The cytotoxicity of LNCaP and Du145 cells was determined using conventional MTT reduction assays. Briefly, cells (4,000 cells/well) were plated in 96-well white-walled, clear-bottom plates (Lonza, Basel, Switzerland) and incubated for 24 h at 37°C. Cells were treated with vehicle (DMSO), increasing concentrations of delphinidin (0 - 90 μM), or increasing concentrations of TRAIL (0, 50, 100, and 150 ng/ml). After 4, 8, and 12 h, 100 μl of assay reagent was added to each well. The plate was incubated in the dark for 15 min, and luminescence was measured using a SpectraMAX 250 (Molecular Device Co., Sunnyvale, CA). All MTT assay data are presented as the mean (± SD) of three independent experiments.

### RNA extraction and quantitative RT-PCR

Total RNA was isolated with the RNA Easyspin kit according to the instructions of the manufacturer (Intron Biotechnology, Seongnam, Korea). Total RNA from each sample was reverse transcribed with random primers using a StrataScript^TM^ reverse transcriptase kit (Qiagen Inc., Valencia, CA) according to the manufacturer's protocol. Quantitative real-time RT-PCR (qRT-PCR) was performed using a SYBR® Premix Ex Taq^TM^ Kit (TaKaRa, Shiga, Japan) with forward and reverse primers for each gene. Primers for amplification of the *DR5* transcript were 5′-TGACGGGGAAGAGGAACTGA-3′ and 5′-GGCTTTGACCATTTGGATTTGA-3′. Primers for *p21*/*CDKN1A* amplification were 5′-GTGGAGAGCATT CCATCCCT-3′ and 5′-TGGATGCAGCTTCCTCT CTG-3′. Primers for *BAX* amplification were 5′-TCTACTTTGCCAGCAAACTGGTGC-3′ and 5′-TGTCCAGCCCATGATGGT TCTGAT-3′. Primers for *BCL2* amplification were 5′-CATGCTGGGG CCGTACAG-3′ and 5′-GAACCGGCA CCTGCACAC-3′. Primers for Survivin amplification were 5′-TGCCTGGCAGCCCTTTC-3′ and 5′-CCTCCA AGAAGGGCCAGTTC-3′. Primers for *MCL1* amplification were 5′-GGGCAGGATTGTGAC TCTCATT-3′ and 5′-GATGCAGCTTTCTTGG TTTATGG-3′. Primers for *XIAP* amplification were 5′-AGTGGTAGTCCTG TTTCAGCATCA-3′ and 5′-CCGCACGGTATCTCCTTCA-3′. Primers for *cIAP2* amplification were 5′-TCC GTCAAGTTCAAGCCAGTT-3′ and 5′-TCTCCTGGGCTGTCTGATGTG-3′. The quantitative real-time PCR was carried out as previously described [74]. Briefly, 1 μl of cDNA from the RT reaction was added to 20 μl of the real-time quantitative polymerase chain reaction mixture containing 10 μl of 2× SYBR^®^ Premix Ex Taq^TM^, and 0.2 μM forward and reverse primers. PCRs were carried out in ABI 7500 Real-Time PCR System (Applied Biosystems, Carlsbad, CA). The samples were incubated at 95°C for 10 min, followed by 40 cycles at 95°C for 30 s and then at 60°C for 1 min. All samples were normalized to human *GAPDH* and expressed as fold induction. All reactions were done in triplicate. Relative expression levels and SDs were calculated using the comparative method.

### siRNA transfection

siRNAs in this study were purchased from Bioneer, Korea and had the following siRNA sequences: siRNA DR5 was 5′-AUCAGCAUCGUGUACAAGGUGUCCC; siRNA BAX was 5′-AAGACCCGCGCCGAGGUGAAG; siRNA HDAC1 was 5′-GAGUCAAAACAGAGGAUGA; siRNA HDAC2 was 5′-GACGGAAACUGAGCUCAGU; siRNA HDAC3 was 5′-GAGCUUCAAUAUCCCUCUA; siRNA HDAC8 was 5′-GUGUCUUAAGUACAUCCUU; siRNA BAX was 5′-GCUGGACAUUGGACUUCCU. LNCaP cells were cultured in each well of 6-well plates for 12 h. For siRNA transfection, Lipofectamine 2000 (Invitrogen) was added to 100 pmol siRNA in a final volume of culture medium. After 36 h of transfection, cells were treated with delphinidin and TRAIL for 12 h.

### DAPI staining

Untreated control cells and cells treated with the delphinidin and/or TRAIL were washed with PBS and fixed with 3.7% paraformaldehyde (Sigma) in PBS for 10 min at room temperature. The fixed cells were washed with PBS and stained with a 1 mg/mL DAPI (Sigma) solution for 10 min at room temperature. The cells were washed two more times with PBS and examined using a fluorescence microscope (BX50; Olympus, Tokyo, Japan).

### HDAC activity assay

A histone deacetylase (HDAC) activity assay was carried out according to the manufacturer's instructions by using an available commercial kit (BioVision Biotechnology, Milpitas, CA). For specific HDAC activity assays, HDAC1, HDAC2, HDAC3, and HDAC8 proteins were immunoprecipitated from LNCaP nuclear extracts by using anti-HDAC1, anti-HDAC2, anti-HDAC3, and anti-HDAC8 antibodies. Immunoprecipitated complexes were collected and washed with an HDAC assay buffer (50 mmol/L Tris pH 8.0, 10% glycerol, 0.1 mmol/L EDTA).

### TUNEL assay

To detect cellular apoptosis DNA fragmentation was evaluated by a TUNEL assay using HT Titer TACS Assay Kit (Trivigen, Gaithersburg, MD, Cat No., 4822-96-K), according to the manufacturer's instructions. Briefly, the cells were fixed with 3.7% buffered formaldehyde solution for 7 min, washed with PBS, permeabilized with 100% methanol for 20 min, washed twice with PBS, digested with proteinase K for 15 min, quenched with 3% hydrogen peroxide, washed with distilled water, labeled with deoxynucleotidyl transferase, incubated at 37°C for 90 min, and treated with stop buffer. The cells were incubated with a TACS-Sapphire substrate, and the colorimetric reaction was stopped with 0.2N HCl after 30 min. The cells were incubated with a TACS-Sapphire substrate, and the colorimetric reaction was stopped with 0.2N HCl after 30 min. The cells were visualized by fluorescence microscopy. TUNEL positive cells were counted in at least five microscopic frames of the sections.

### Statistical analyses

Statistical analysis was performed using Student's *t*-tests and the SPSS program (SPSS Inc., Chicago, IL). A statistical threshold of *P* < 0.05 was considered statistically significant.
